# Lifestyle Leaders’ Experiences in Senior Housing During the COVID-19 Pandemic

**DOI:** 10.1093/geroni/igab046.1072

**Published:** 2021-12-17

**Authors:** Kathryn Chan, Taylor Patskanick, Julie Miller

**Affiliations:** 1 Massachusetts Institute of Technology AgeLab, Cambridge, Massachusetts, United States; 2 MIT, Somerville, Massachusetts, United States; 3 MIT, Cambridge, Massachusetts, United States

## Abstract

Millions of older adults living in close communal contact in senior housing communities remain vulnerable during the COVID-19 pandemic. Approximately 30% of Lifestyle Leaders currently live in senior housing. This presentation will cover the unique challenges these participants have encountered, including experiences with and the impact of changing norms and pandemic-related policies within communities over time. In March 2020, 75% of Lifestyle Leaders rated the response of their senior housing community to COVID-19 as “Excellent” or “Very Good.” In August 2020, they reported they believed they were less likely to contract COVID-19 living in senior housing compared to people not living in senior housing (80%). Interview data revealed Lifestyle Leaders in these environments held favorable views toward their communities. This presentation will further discuss how the Lifestyle Leaders who do not live in senior housing perceive senior living and how these perceptions have shifted during the pandemic.

